# Intracellular targeting of annexin A2 inhibits tumor cell adhesion, migration, and *in vivo* grafting

**DOI:** 10.1038/s41598-017-03470-w

**Published:** 2017-06-26

**Authors:** Daniela I. Staquicini, Roberto Rangel, Liliana Guzman-Rojas, Fernanda I. Staquicini, Andrey S. Dobroff, Christy A. Tarleton, Michelle A. Ozbun, Mikhail G. Kolonin, Juri G. Gelovani, Serena Marchiò, Richard L. Sidman, Katherine A. Hajjar, Wadih Arap, Renata Pasqualini

**Affiliations:** 1 University of New Mexico Comprehensive Cancer Center, and Division of Molecular Medicine, Department of Internal Medicine, University of New Mexico School of Medicine, Albuquerque, NM 87131 USA; 2 Department of Immunology, The University of Texas M.D. Anderson Cancer Center, Houston, TX 77054 USA; 3 Cancer Research Program, Houston Methodist Research Institute, Houston, TX 77030 USA; 4 Department of Molecular Genetics and Microbiology, University of New Mexico School of Medicine, Albuquerque, NM 87131 USA; 5 Institute of Molecular Medicine, University of Texas Health Science Center at Houston, Houston, TX 77030 USA; 6 Department of Biomedical Engineering, College of Engineering and School of Medicine, Wayne State University, Detroit, MI 48202 USA; 7 Department of Oncology, University of Turin, Candiolo, TO 10060 Italy; 8 Candiolo Cancer Institute – FPO, IRCCS, Candiolo, TO 10060 Italy; 9 Department of Neurology, Harvard Medical School, Beth Israel Deaconess Medical Center, Boston, MA 02215 USA; 10 Departments of Pediatrics, Cell and Developmental Biology, and Medicine, Weill Cornell Medicine College, New York, NY 10065 USA; 11 University of New Mexico Comprehensive Cancer Center, and Division of Hematology/Oncology, Department of Internal Medicine, University of New Mexico School of Medicine, Albuquerque, NM 87131 USA

## Abstract

Cytoskeletal-associated proteins play an active role in coordinating the adhesion and migration machinery in cancer progression. To identify functional protein networks and potential inhibitors, we screened an internalizing phage (iPhage) display library in tumor cells, and selected LGRFYAASG as a cytosol-targeting peptide. By affinity purification and mass spectrometry, intracellular annexin A2 was identified as the corresponding binding protein. Consistently, annexin A2 and a cell-internalizing, penetratin-fused version of the selected peptide (LGRFYAASG-pen) co-localized and specifically accumulated in the cytoplasm at the cell edges and cell-cell contacts. Functionally, tumor cells incubated with LGRFYAASG-pen showed disruption of filamentous actin, focal adhesions and caveolae-mediated membrane trafficking, resulting in impaired cell adhesion and migration *in vitro*. These effects were paralleled by a decrease in the phosphorylation of both focal adhesion kinase (Fak) and protein kinase B (Akt). Likewise, tumor cells pretreated with LGRFYAASG-pen exhibited an impaired capacity to colonize the lungs *in vivo* in several mouse models. Together, our findings demonstrate an unrecognized functional link between intracellular annexin A2 and tumor cell adhesion, migration and *in vivo* grafting. Moreover, this work uncovers a new peptide motif that binds to and inhibits intracellular annexin A2 as a candidate therapeutic lead for potential translation into clinical applications.

## Introduction

Cell adhesion and migration require dynamic remodeling of the cytoskeleton. This process results from the coordinated activity of several proteins, among which members of the annexin family of calcium- and phospholipid-binding proteins^1, 2^. Annexins are involved in a variety of processes including membrane organization, intracellular trafficking, and cytoskeleton remodeling in normal and diseased tissues^3–5^. In vertebrates, annexins are grouped into 12 subfamilies that share a basic structural core composed of four annexin repeats (eight in annexin A6) mediating reversible calcium-dependent binding to biological membranes, and a variable N-terminal domain responsible for protein-protein interactions^4^. In addition, annexins 1 and 2 include phosphorylation domains for different signal transducing kinases, as well as binding sites for the calcium-binding proteins S100A10 and S100A11. Annexin A2 is anchored at the plasma membrane as a heterotetrameric complex with S100A10^6^. This complex interacts with cytoskeleton components such as filamentous actin (F-actin) in the assembly of dynamic structures during phagocytosis, pinocytosis and cell migration^3, 7^.

Clinical studies have shown that annexin A2 is highly expressed in different tumor types, including gastric, colorectal, pancreatic, breast, and kidney cancers, high-grade gliomas, along with vascular tumors^8–12^. Preclinical studies have revealed a functional role for extracellular annexin A2 in the regulation of adhesion, migration, homing, and invasion of cancer cells^13–16^. Several annexin A2-interacting proteins, e.g. epithelial growth factor receptor (EGFR)^17^, migration and invasion enhancer 1 (MIEN1)^16^, galectin-3^15^, and β1 integrin^18^, have been described to mediate tumor progression through phosphorylation and translocation of annexin A2 to the cell surface. Extracellular annexin A2, in association with S100A10, regulates the proteolytic activity of plasmin, leading to hydrolysis and remodeling of the extracellular matrix (ECM) and activation of matrix metalloproteases in tumor invasion^19, 20^. Although annexin A2 has been extensively studied as a component of supramolecular complexes at the cell surface, it is also abundant as a cytosolic monomer. However, its role as an intracellular protein in cancer progression is not well understood.

We have recently designed and validated internalizing iPhage random peptide display libraries, an enabling platform based on viral particles that can be delivered intracellularly by exploiting the receptor-independent internalization of a penetratin (pen) moiety fused to the major capsid protein. This combinatorial approach allowed the identification and characterization of motifs targeting certain organelles and their molecular pathways within live cells^21, 22^. Here we report the discovery of an annexin A2 targeting motif, LGRFYAASG, identified by screening an iPhage library in KS1767, a human Kaposi’s sarcoma-derived cell line. A synthetic cell-penetrating version of this peptide (LGRFYAASG-pen) interacts with intracellular annexin A2 and disrupts F-actin and focal adhesions, thus impacting on tumor cell shape and impairing their attachment to the ECM. At the molecular level, tumor cells incubated with LGRFYAASG-pen show decreased phosphorylation of Fak and Akt, indicating a specific involvement of focal adhesion-associated annexin A2. The intracellular targeting of annexin A2 also reduces caveolae-related trafficking, supporting an effect on lipid raft stability and cell signaling. Finally, LGRFYAASG-pen inhibits tumor cell migration *in vitro* and reduces the formation of experimental lung colonies *in vivo*. Together, our results provide mechanistic insights into the association of intracellular annexin A2 with the cytoskeletal machinery in tumor cells, and exploit its role as an intracellular target in cancer progression.

## Results

### The iPhage-displayed LGRFYAASG peptide serves as intracellular ligand for annexin A2

**Figure 1:**
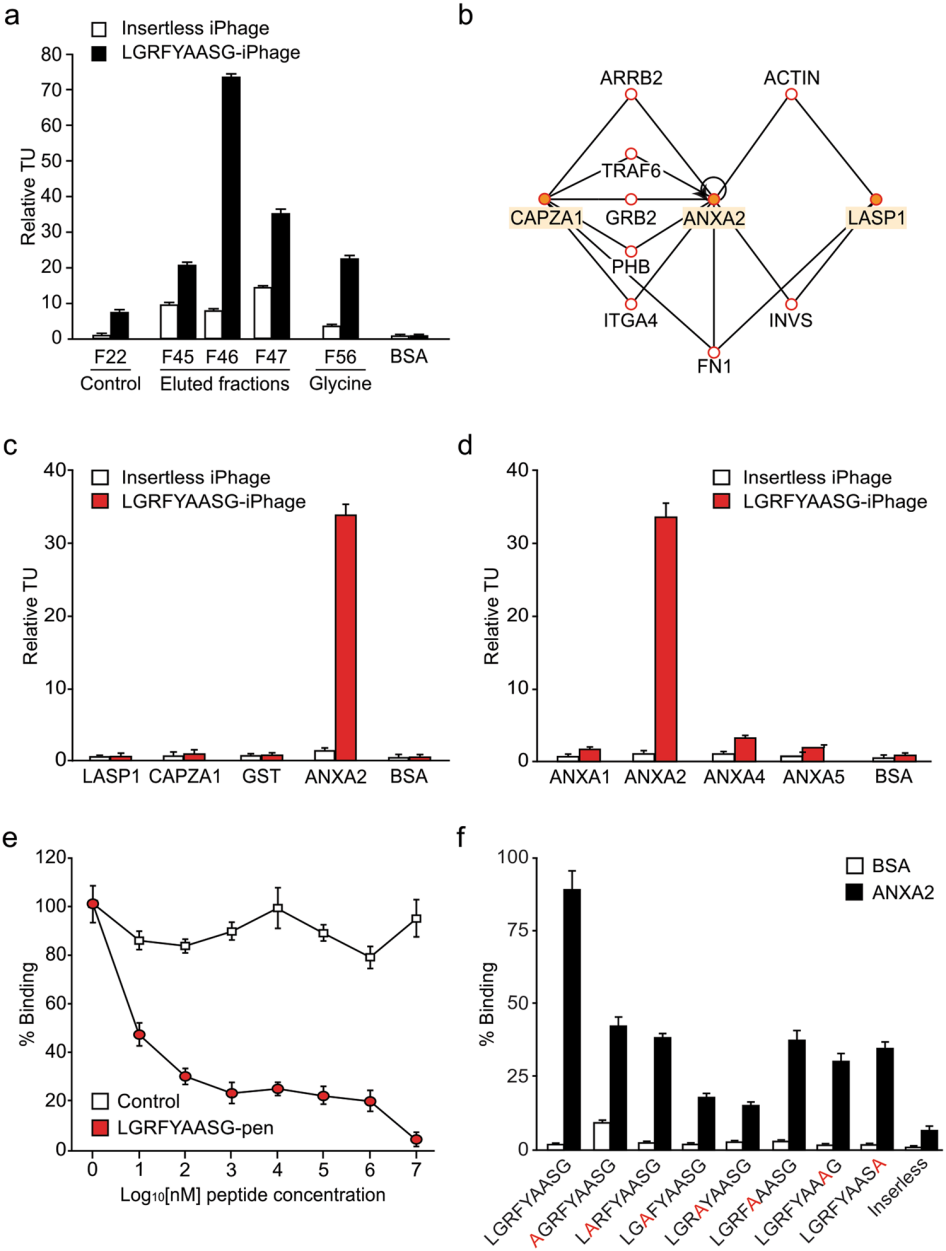
The LGRFYAASG peptide motif is a specific intracellular ligand for annexin A2. (**a**) iPhage binding assays on protein fractions obtained by affinity chromatography. BSA was used as a negative protein control. TU values were normalized on binding of the insertless iPhage to annexin A2. (**b**) *In silico* analysis of potential interactions among the mass spectrometry-identified proteins in the canonical actin cytoskeleton pathway, as determined by the IPA protein network prediction software. ANXA2, annexin A2. (**c**) Binding of iPhage to GST-fused recombinant annexin A2, LASP1, and CAPZA1. GST and BSA served as protein controls. (**d**) Binding of LGRFYAASG-iPhage to recombinant annexin A1 (ANXA1), A2 (ANXA2), A4 (ANXA4), and A5 (ANXA5). (**e**) Binding of LGRFYAASG-iPhage to annexin A2 in the presence of increasing concentrations of the synthetic peptide LGRFYAASG-pen. Unconjugated pen was used as a control, and binding in the absence of the inhibiting peptide was set at 100%. (**f**) Binding of alanine scanning variants of LGRFYAASG-iPhage. TU values were normalized on binding of an insertless iPhage, and bars represent mean values of triplicate experimental points ± standard error of the mean (SEM).

To identify cell-penetrating agents targeting intracellular proteins, we screened a random iPhage peptide library in cultured KS1767 Kaposi’s sarcoma cells. After overnight incubation, iPhage clones were collected from the cytosol fraction of whole cell lysates, recovered and amplified by *E. coli* infection, and purified for successive selection rounds. After five rounds of synchronous selection, the LGRFYAASG motif was specifically enriched and further investigated. By solid-phase (Merrifield) synthesis, a cell-internalizing version of the corresponding soluble peptide was generated *via* C-terminal fusion to the pen motif. Affinity chromatography served to purify the intracellular protein binding partner(s) for LGRFYAASG-pen in KS1767 cell lysates. Eluted fractions were immobilized in 96-well plates and phage binding assays revealed high concentrations of potential interactors in fractions F45–47 (Fig. 1a). Proteins with molecular weights of 33, 36 and 38 kDa were recovered from fraction F46 and analyzed by mass spectrometry, leading to the identification of three candidates: F-actin capping protein alpha-1 subunit (CAPZA1), Lim SH3 protein 1 (LASP1), and annexin A2 (Table S1). A bioinformatic analysis suggested that CAPZA1, LASP1 and annexin A2 interact through a network of proteins, some of which are related to cytoskeleton dynamics, cell adhesion and migration in cancer, namely fibronectin^23^, α-actin^24^, and growth factor receptor-bound protein 2 (Grb2)^25, 26^ (Fig. 1b). We tested the interaction of the LGRFYAASG-displaying iPhage with each candidate recombinant protein, and observed specific binding to annexin A2, but not to either LASP1 or CAPZA1 (Fig. 1c). Other proteins of the family (annexins A1, A4, and A5) showed background binding only (Fig. 1d). Binding of LGRFYAASG-iPhage to annexin A2 was inhibited in a concentration-dependent manner by the synthetic peptide LGRFYAASG-pen, indicating a specific saturable interaction (Fig. 1e). Alanine-scanning mutagenesis of the iPhage-displayed LGRFYAASG sequence identified the arginine (R^3^) and phenylalanine (F^4^) residues as critical for binding to annexin A2 (Fig. 1f). Together, our findings confirm LGRFYAASG-pen as a specific peptide ligand for annexin A2.

### LGRFYAASG-pen disassembles focal adhesions, actin filaments and lipid microdomains

**Figure 2:**
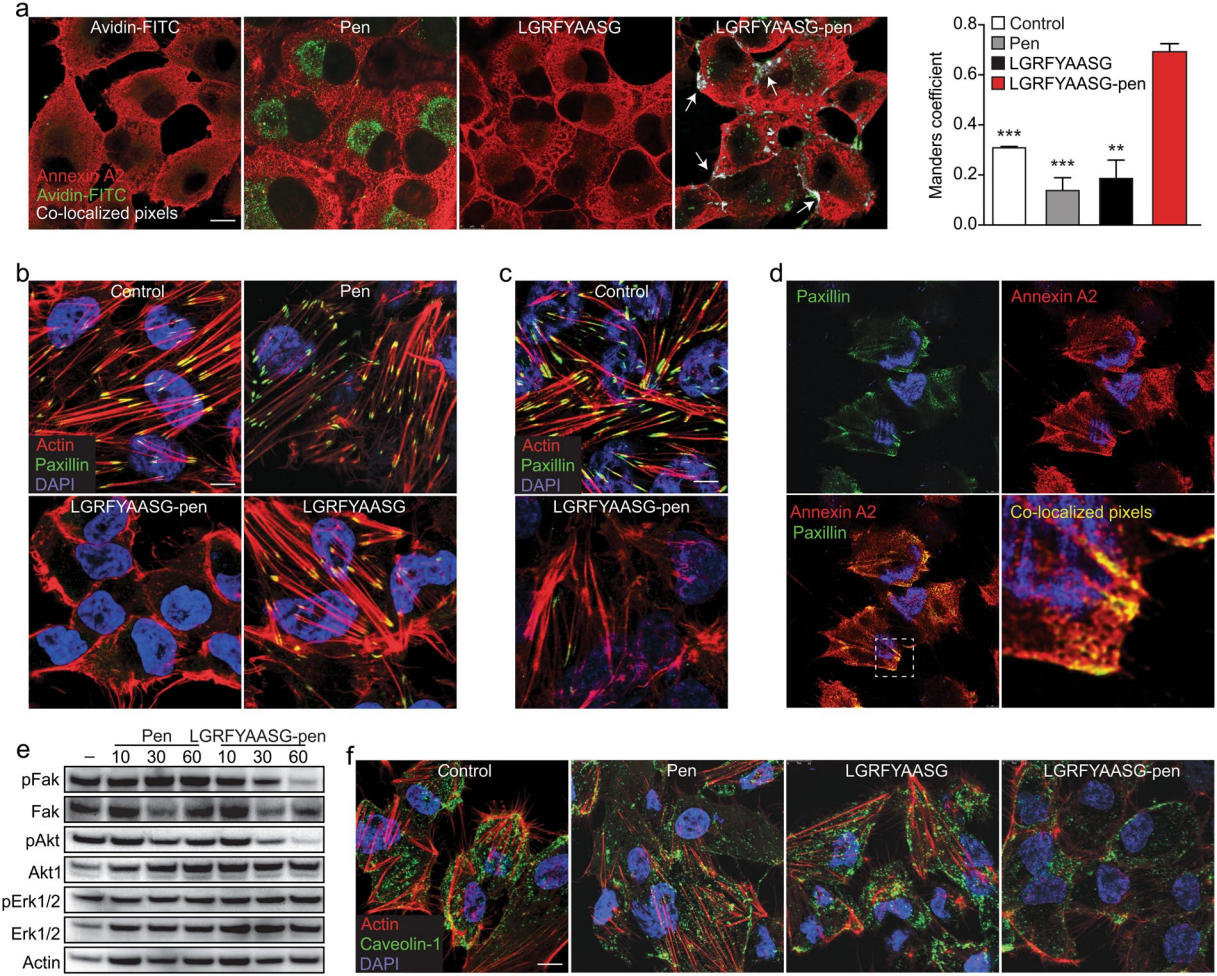
LGRFYAASG-pen co-localizes with annexin A2 and disrupts actin filaments, focal adhesions and caveolin-rich microdomains. (**a**) Co-localization analysis: KS1767 cells grown overnight onto circular coverslips were incubated for 2 h at 37 °C with biotinylated LGRFYAASG-pen, or unconjugated LGRFYAASG (10 µM). Untreated cells were used as controls. IF staining of biotinylated peptides (avidin-FITC, green) and annexin A2 (Cy3-conjugated secondary antibody, red) was analyzed with Fiji ImageJ. Co-localized pixels are visualized in white (arrows), and Manders’ overlap coefficients are reported in the graph (**P < 0.01; ***P < 0.001 *versus* LGRFYAASG-pen). (**b**,**c**) Disassembly of actin filaments: IF staining of paxillin (FITC-conjugated secondary antibody, green) and actin (rhodamine-phalloidin, red) in KS1767 cells grown onto (**b**) fibronectin/vitronectin- or (**c**) type I collagen-coated coverslips. (**d**) Localization of annexin A2 at the focal adhesions: KS1767 cells co-stained for paxillin (FITC-conjugated secondary antibody, green) and annexin A2 (Alexa 647-conjugated secondary antibody, red). An enlarged inset (white dotted line) is included for prompt visualization. (**e**) Western blot of Fak, Akt and Erk1/2 phosphorylation following KS1767 cell incubation with pen only or LGRFYAASG-pen for the indicated periods of time. Total Fak, Akt, Erk1/2 were used as a reference, and actin served as loading control. (**f**) Disruption of caveolin-rich microdomains: KS1767 cells incubated with the peptides (30 µM) followed by IF staining of caveolin-1 (FITC-conjugated secondary antibody, green) and actin (red). In (**b**), (**c**), (**d**), (**f**), nuclei are visualized with DAPI (blue); in all micrographs: scale bar, 10 µm.

To gain mechanistic insight, we investigated the intracellular interaction between the targeting peptide and annexin A2 by confocal immunofluorescence (IF) microscopy. A biotin-conjugated version of either LGRFYAASG-pen or control peptides (pen only, unconjugated LGRFYAASG) was incubated for 2 h with cultured KS1767 cells, and successively visualized with avidin-fluorescein isothiocyanate (FITC). In these assays, a cytoplasmic co-localization of LGRFYAASG-pen and annexin A2 was observed in discontinuous cellular structures, particularly at the cell edges and at sites of cell-cell interaction (Fig. 2a, rightmost microphotograph). In contrast, neither avidin-FITC nor the unconjugated peptide accumulated within the cells. Detectable amounts of pen and LGRFYAASG-pen were visible in intracellular vesicles with the structure of lysosomes (Fig. 2a, green spots), suggesting that some excess peptide might have entered this degradation pathway. Cytoplasmic annexin A2 has been shown associated to the actin cytoskeleton^27, 28^, as well as to the acidic phospholipids present in specific membrane microdomains^29, 30^. The peculiar co-localization of LGRFYAASG-pen and annexin A2 (Fig. 2a) was somehow reminiscent of such cell compartments, and suggested that interfering with intracellular annexin A2 might impact on their appearance, integrity and/or functionality. To test this working hypothesis, we first evaluated the actin-based structures and membrane-cytoskeleton linkages. KS1767 cells were seeded on an admixture of fibronectin and vitronectin, and incubated overnight with LGRFYAASG-pen or control peptides. Staining for actin and paxillin^31^ revealed disassembly of F-actin and disappearance of focal adhesions, respectively, in cells treated with the intracellular annexin A2-targeting peptide, but not with control peptides including the non-internalizing LGRFYAASG (Fig. 2b). A similar outcome was obtained with cells seeded on type I collagen (Fig. 2c), demonstrating that this mechanism is independent of the ECM composition. In support of these findings, specific co-localization of annexin A2 and paxillin was detected in KS1767 cells under the same experimental conditions (Fig. 2d). The unambiguous involvement of annexin A2 in the maintenance of cell structure integrity was further confirmed by silencing annexin A2 with specific lentiviral shRNA clones (Fig. S1a). KS1767 cells with silenced expression of annexin A2 exhibited the same disruption of F-actin and focal adhesions observed upon LGRFYAASG-pen treatment (compare Fig. 2b,c with GFP-positive cells in Fig. S1b,c). At the molecular level, incubation of KS1767 cells with LGRFYAASG-pen resulted in a time-dependent decreased phosphorylation of the focal adhesion-associated kinases Fak^32^ (Tyr^397^) and Akt (Ser^473^), while no inhibitory effect was observed on mitogen-activated kinase Erk1/2 (p42/p44, Thr^202^/Tyr^204^) (Fig. 2e). We next investigated the effect of LGRFYAASG-pen on KS1767 cell membrane organization, in particular on phosphatidyl inositol 4,5-bisphosphate-, cholesterol- and glycosphingolipid-rich microdomains defined as lipid rafts or caveolae^33^. Staining of caveolin-1 revealed disruption of caveolae in the presence of LGRFYAASG-pen (but not of control peptides), indicative of an impaired membrane trafficking (Fig. 2f). Together, these results show that the intracellular annexin A2-targeting peptide inhibits annexin A2-driven modulation of actin cytoskeleton, focal adhesions and lipid microdomains, with repercussion on cell signaling.

### LGRFYAASG-pen inhibits tumor cell adhesion and migration

**Figure 3:**
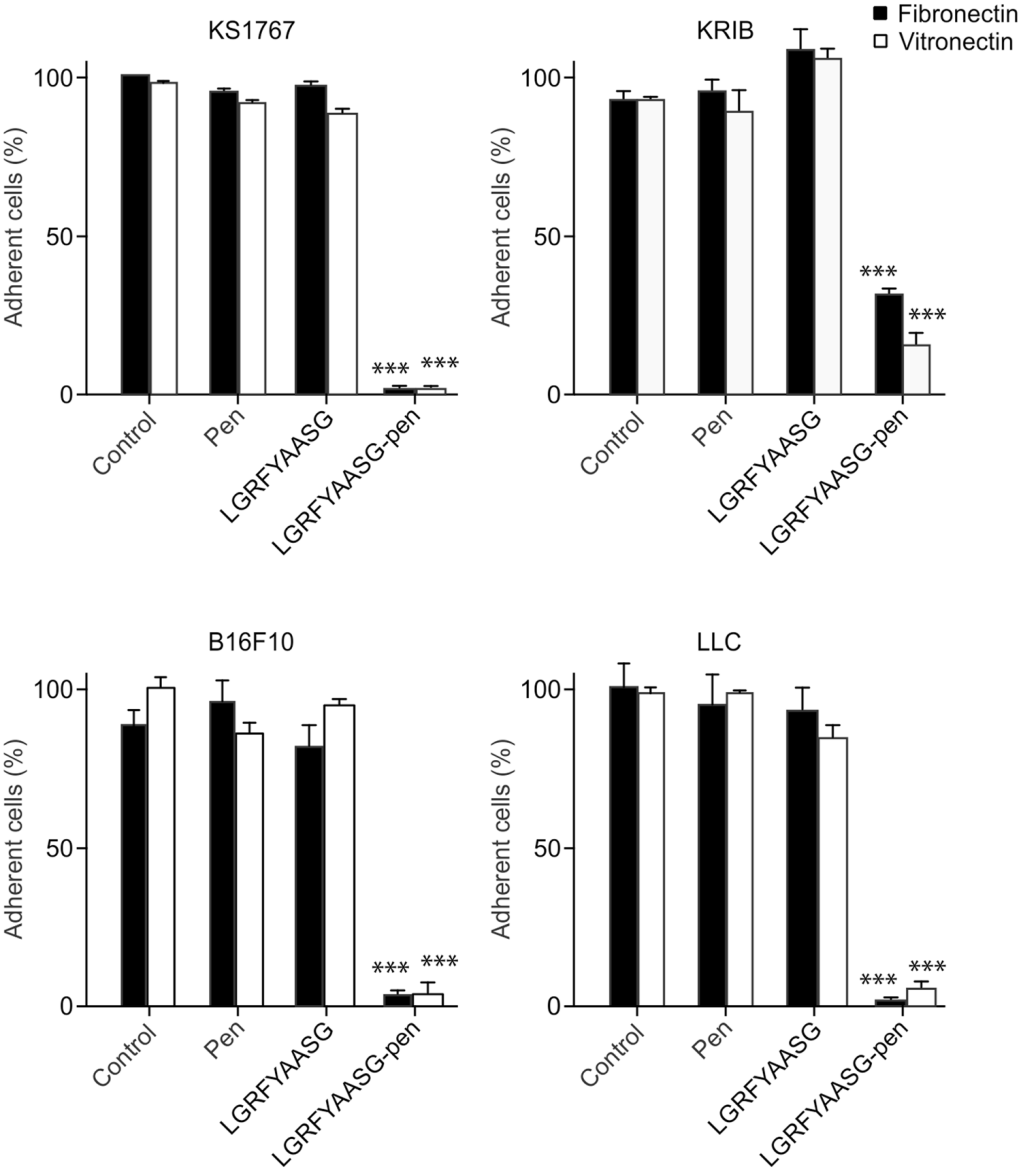
LGRFYAASG-pen inhibits adhesion of tumor cells. Adhesion of KRIB, KS1767, B16F10 and LLC cells to fibronectin or vitronectin in the presence of LGRFYAASG-pen (30 µM). Unconjugated LGRFYAASG and pen served as negative controls (***P < 0.0001 *versus* control experimental points).

**Figure 4:**
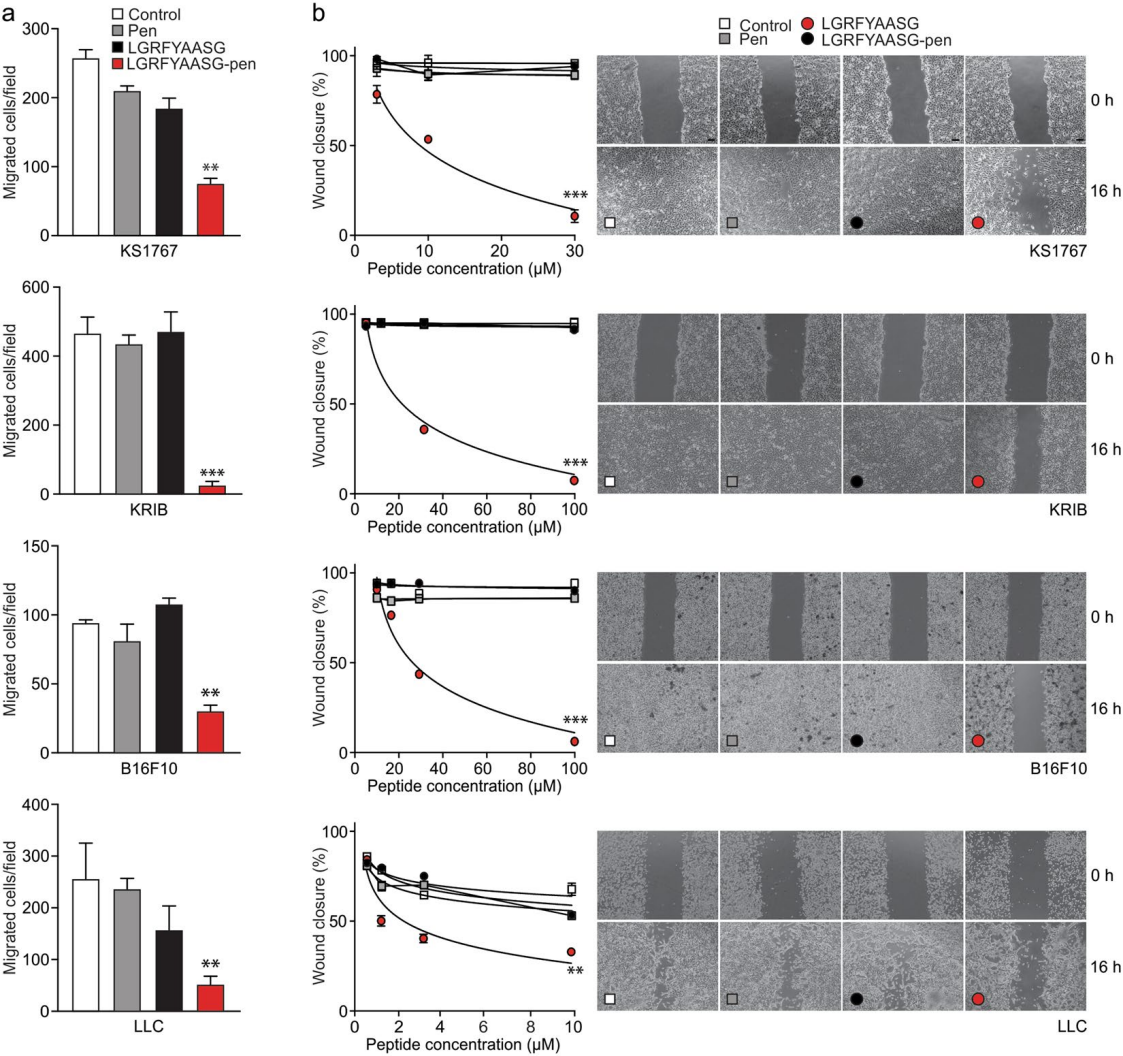
LGRFYAASG-pen inhibits tumor cell migration. (**a**) Transwell cell migration assays: 10^5^ cells were applied to the upper chamber of transwell inserts in the presence of each peptide (100 µM for KS1767, KRIB, B16F10, 10 µM for LCC). After 24 h, migrated cells were fixed, stained in crystal violet and counted (5 fields/well) under a light microscope (**P < 0.001; ***P < 0.0001 *versus* the control experimental points). (**b**) Wound-healing cell motility assays performed with KS1767, KRIB, LLC and B16F10 cells. A wound was produced with a pipetman tip in confluent cell layers, followed by incubation with either medium only or the indicated peptides (3–30 μM for KS1767, 10–100 μM for KRIB and B16F10, 1–10 μM for LLC). The width of the wounds was imaged with a phase-contrast microscope, and is represented as percent of wound closure. Micrographs refer to the highest peptide concentrations. Scale bar, 100 µm. (**P < 0.001; ***P < 0.0001 *versus* control experimental points).

To further validate the biological effect of LGRFYAASG-pen, we performed adhesion assays with KS1767 human Kaposi’s sarcoma, KRIB human osteosarcoma, B16F10 murine melanoma and Lewis lung mouse carcinoma (LLC) cell lines on fibronectin- or vitronectin-coated wells. In preliminary assays, increasing concentrations of LGRFYAASG-pen progressively inhibited KS1767 cell attachment, whereas no detectable effect was observed with controls, including either pen alone or the non-internalizing LGRFYAASG peptide (Fig. S2a). A specific inhibition of cell adhesion by LGRFYAASG-pen was observed in the complete cell panel, and was independent of tumor type (sarcoma, melanoma or carcinoma), species origin (human or murine), and adhesive matrix (fibronectin or vitronectin) (Fig. 3), suggesting an overall impairment of the cytoskeleton. We next tested the same cell lines in transwell (Fig. 4a) and wound-healing (Fig. 4b) migration assays. In both cases, a specific inhibition was observed for all tumor cell lines in the presence of LGRFYAASG-pen compared to the controls. In the *in vitro* wound-healing assays, migration of LLC cells was inhibited at lower molar concentrations (3–10 μM), while KS1767, KRIB and B16F10 cells were inhibited at higher molar concentrations (100 μM) (Fig. 4b). Cells remained viable, and grew with an unaltered kinetics for up to 96 h at the highest concentrations of either LGRFYAASG-pen or control peptides, ruling out non-specific toxicity as a cause of the decreased adhesion (Fig. S2b). Together, these data show that LGRFYAASG-pen impairs tumor cell adhesion and migration, likely by inhibiting annexin A2-dependent membrane-cytoskeleton attachment and organization of actin-based structures.

### LGRFYAASG-pen impairs experimental tumor engrafting *in vivo*

**Figure 5:**
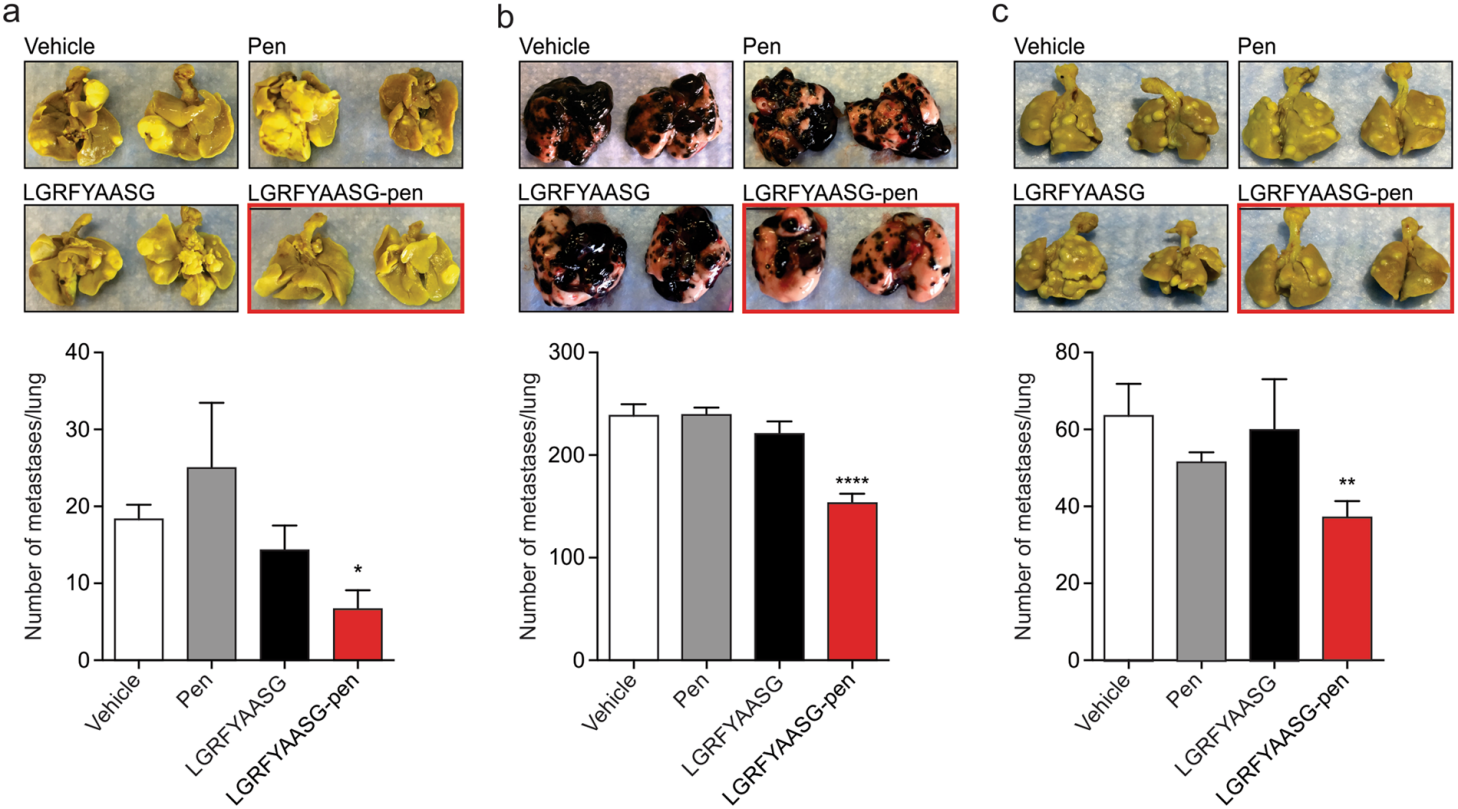
LGRFYAASG-pen inhibits the growth of experimental tumors in several preclinical models. Representative gross morphology of lungs explanted after 8 weeks (KRIB, (**a**), 21 days (B16F10, (**b**), 13 days (LLC, (**c**) after fixation in Bouin’s fixative, and corresponding lung weights (n = 8 animals/group) shown as mean ± SEM (****P < 0.0001, **P < 0.001, *P < 0.01 *versus* vehicle experimental points).

Having demonstrated that LGRFYAASG-pen alters the adhesion and migration of cancer cells *in vitro*, we asked whether it would also affect the onset of experimental tumors *in vivo*. For this purpose, we chose a well-characterized model of lung colonization that provides an optimal short time-frame window for evaluating effects on tumor growth. The *in vivo* highly aggressive KRIB (Fig. 5a), B16F10 (Fig. 5b), or LLC (Fig. 5c) cells were incubated for 30 min with either LGRFYAASG-pen or control peptides prior to intravenous administration in mice. LGRFYAASG-pen-treated cells showed diminished colonization of the lungs, as evidenced by significantly fewer tumor foci compared to control-incubated cells. The lungs were pathologically evaluated by hematoxylin and eosin (H&E) staining, which confirmed that fewer and smaller microscopic foci developed from tumor cells pre-treated with LGRFYAASG-pen (Fig. S3). These findings demonstrate that presumably the cell-internalizing LGRFYAASG-pen peptide impairs lung colonization by tumor cells in preclinical models by interfering with annexin A2-mediated cell adhesion and/or migration.

## Discussion

Here we identify and characterize the LGRFYAASG peptide as a targeting moiety that binds to intracellular annexin A2 and interferes with tumor cell adhesion and migration *in vitro*, resulting in impaired tumor grafting *in vivo*. Given the high expression of annexin A2 in many human tumors^8–12^, this discovery represents a promising step towards potential therapeutic intervention.

Annexin A2 is a pleiotropic protein involved in a wide range of molecular and cellular processes that converge on the regulation of cell shape, adhesion, and motility. The participation of extracellular annexin A2 has been widely described in cell migration^18, 34–36^. In particular, many studies suggest that annexin A2-mediated tumor cell invasiveness is attributable to the generation of extracellular plasmin through modulation of S100A10 enzyme activity. In our findings, we show that the intracellular pool of annexin A2 also plays an important role as a regulator of tumor cell adhesion, migration, and, potentially, secondary dissemination. Annexin A2 includes binding sites for calcium, actin and acidic phospholipids, which are responsible for its dynamic accumulation in regions of membrane-cytoskeleton connection^3, 7^, and in phosphatidyl inositol 4,5-bisphosphate-, cholesterol-, and glycosphingolipid-rich lipid rafts^34, 35^. Consistently, we show that LGRFYAASG-pen binds to intracellular annexin A2 at discontinuous membrane domains, potentially disrupting its activity at these sites. In detail, we demonstrate that the intracellular annexin A2-targeting peptide LGRFYAASG-pen promotes disorganization of F-actin, disassembles focal adhesions, and reduces the number of lipid raft-associated trafficking vesicles.

Notably, the LGRFYAASG peptide sequence is a mimic of Cdc42, a member of the Rho family of GTPases (residues 53–60 of the human protein, UniProt entry P60953). This portion of Cdc42 is exposed when the protein is bound, e.g., to the serine/threonine-protein kinase PAK 1 (molecular modeling database, MMDB ID:12918) or Wiskott–Aldrich syndrome protein (WASP, MMDB ID:10496) during actin remodeling and filopodia formation^37^. The external location of this motif in the tertiary structure of Cdc42 suggests its physical accessibility as protein-protein recognition site. These observations are consistent with previous data indicating a direct interaction between annexin A2 and Cdc42 in epithelial polarization, and *de novo* apical domain and lumen formation^38^, and thereby support the hypothesis that intracellular LGRFYAASG might act as a competitive inhibitor of the annexin A2/Cdc42 complex. Future research including X-ray crystallography studies might shed more light into this intriguing observation.

In addition to annexin A2, two other candidate proteins, CAPZA1 and LASP1, were enriched by affinity chromatography against LGRFYAASG-pen, but specific binding assays with LGRFYAASG-displaying iPhage particles demonstrated that they are not direct interactors of this peptide. Such proteins might bind the peptide indirectly, by taking part in a supramolecular complex that includes annexin A2. Consistently, our bioinformatic analysis revealed that annexin A2, CAPZA1 and LASP1 indeed share common interactors. Among these, fibronectin 1^23^, α-actin^24^, and Grb2^25, 26^ are well-characterized in several signaling networks in cancer settings. Other interacting members, such as arrestin β2 (ARRB2)^39^, tumor necrosis factor (TNF) receptor associated factor 6 (TRAF6)^40^, prohibitin (PHB)^3–5, 41^, integrin α4 (ITGA4)^42^, and inversin (INVS)^43^ have been recently and/or marginally related to cancer cell motility and invasion. These data support the action of functional supramolecular complexes, rather than a single protein species, in mediating adhesion and motility during cancer progression. As such, these integrated networks might be therapeutically targeted with potentially increased efficacy, for example by LGRFYAASG-pen, compared, e.g., to a classic small-molecule inhibitor targeting a single protein.

In conclusion, here we show that a prototype peptide such as cell-internalizing LGRFYAASG-pen mechanistically interferes with intracellular annexin A2, representing a multifaceted inhibitor of cytoskeletal arrangements pivotal for key steps in cancer progression in preclinical tumor models. The potential for translation of these initial observations into clinical applications remains an open avenue for future studies.

## Materials and Methods

### Cells and reagents

KS1767, KRIB, B16F10 and LLC cells (ATCC) were maintained in Dulbecco’s modified Eagle’s Minimum Essential Medium (DMEM) supplemented with 10% fetal bovine serum (FBS), vitamins, non-essential amino acids, penicillin/streptomycin, and L-glutamine (Gibco) at 37 °C in a 5% CO_2_ humidified incubator. The 4′,6-diamidino-2-phenylindole (DAPI) nuclear stain was from Vector Laboratories. Human recombinant annexin A1, annexin A2, annexin A4 and annexin A5 were from Amprox; fibronectin and vitronectin were from R&D Systems; type I collagen I was from ScienCell Research Laboratories. Recombinant GST was produced in *E. coli* transformed with pGEX4T-1 plasmid (Amersham) and purified with standard protocols. All synthetic peptides were commercially manufactured and quality-controlled to our specifications (PolyPeptide Laboratories).

### iPhage library intracellular selection

KS1767 cells were incubated with 5 × 10^11^ transducing units (TU) of the X_4_YX_4_ (X, any residue; Y, tyrosine) iPhage library overnight at 37 °C, extensively washed with pre-warmed PBS and subsequently detached with trypsin. Cell suspensions were washed with ice-cold PBS, incubated in hypotonic buffer (10 mM NaCl, 1.5 mM MgCl_2_, 10 mM Tris-HCl pH 7.5) for 15 min, and disaggregated in a standard Dounce homogenizer. A stabilization buffer was added (final concentration: 84 mM mannitol, 70 nM sucrose, 1 mM EDTA pH 7.5, 5 mM Tris-HCl pH 7.5), and the resulting organelle suspension was centrifuged at 1,300 g for 5 min at 4 °C to enrich for the nuclear fraction. The supernatant was recovered and centrifuged at 17,000 g for 15 min; this pellet contained the mitochondrial/ER fraction. Finally, the remaining supernatant was centrifuged at 100,000 g for 40 min to obtain the cytosolic fraction. The cytosol-retained phage population was recovered, amplified and purified as described^44^. After five rounds of selection, 96 bacterial colonies from each fraction were randomly picked for DNA sequencing.

### Affinity purification and identification of intracellular binding partners of LGRFYAASG

To isolate specific intracellular interactors, LGRFYAASG-pen was coupled to Sepharose columns (CarboxyLink Immobilization kit, Pierce) and incubated with KS1767 protein extracts. After extensive washing in phosphate buffered saline (PBS), bound proteins were eluted with the corresponding soluble peptide (5 mM). Eluted fractions were analyzed by absorbance at 280 nm, dialyzed and concentrated at 4 °C. Total protein (5 µg/well in 50 µl PBS) was immobilized onto 96-well plates overnight at 4 °C. Wells were washed twice in PBS, blocked in PBS containing 2% BSA for 2 h at room temperature, and incubated with LGRFYAASG iPhage or insertless iPhage (10^9^ TU) in 50 µl PBS with 0.1% BSA. After 2 h at room temperature, wells were washed ten times with PBS, and phage particles were recovered and quantified by bacterial infection. For the identification of bound proteins, the eluate from F46 was subjected to SDS-PAGE and specific bands were characterized with a Nano LC-MS/MS peptide sequencer (ProtTech) as described^45^.

### Alanine-scanning mutagenesis of iPhage-displayed peptides

Mutant phage particles displaying alanine-scanning variants of LGRFYAASG were prepared by site-directed mutagenesis^46^. Briefly, the following oligonucleotide pairs (Sigma-Genosys) were annealed in 10 mM Tris-HCl pH 8.0, containing 100 mM NaCl, 1 mM EDTA at 10 nM, and cloned into *SfiI*-digested fUSE5 vector with T4 DNA ligase (Roche):

**A**GRFYAASG -sense, 5′- GCGGGCCGCTTTTATGCGGCGAGCGGC-3′, and

**A**GRFYAASG -antisense, 5′-GCCGCTCGCCGCATAAAAGCGGCCCGC-3′;

L**A**RFYAASG -sense, 5′-CTGGCGCGCTTTTATGCGGCGAGCGGC-3′, and

L**A**RFYAASG -antisense, 5′-GCCGCTCGCCGCATAAAAGCGCGCCAG-3′

LG**A**FYAASG -sense, 5′-CTGGGCGCGTTTTATGCGGCGAGCGGC-3′, and

LG**A**FYAASG -antisense, 5′-GCCGCTCGCCGCATAAAACGCGCCCAG-3′;

LGR**A**YAASG -sense, 5′-CTGGGCCGCGCGTATGCGGCGAGCGGC-3′, and

LGR**A**YAASG -antisense, 5′-GCCGCTCGCCGCATACGCGCGGCCCAG-3′

LGRF**A**AASG -sense, 5′-CTGGGCCGCTTTGCGGCGGCGAGCGGC-3′, and

LGRF**A**AASG -antisense, 5′-GCCGCTCGCCGCCGCAAAGCGGCCCAG-3′;

LGRFYAA**A**G -sense, 5′-CTGGGCCGCTATGCGGCGGCGGGC-3′, and

LGRFYAA**A**G -antisense, 5′-GCCCGCCGCCGCATAGCGGCCCAG-3′;

LGRFYAAS**A** -sense, 5′-CTGGGCCGCTTTTATGCGGCGAGCGCG-3′, and

LGRFYAAS**A** -antisense, 5′-CGCGCTCGCCGCATAAAAGCGGCCCAG-3′.

iPhage binding assays were performed with an input of 10^9^ TU/well in 96-well plates and bound phage particles were quantified by bacterial infection.

### Confocal microscopy imaging

Cells were seeded onto circular coverslips in 24-well plates (1.5 × 10^5^ cells/coverslip) in complete medium and grown overnight at 37 °C in 5% CO_2_. For peptide/annexin A2 co-localization studies, adhered cells were pretreated with biotinylated pen, LGRFYAASG or LGRFYAASG-pen (10 µM in complete medium) for 2 h at 37 °C. To evaluate F-actin organization, focal adhesions, and caveolae, the coverslips were pre-coated with either fibronectin/vitronectin or collagen I, and cell pretreatment with the indicated peptides was extended overnight. In all cases, cells were then fixed in 4% paraformaldehyde in PBS for 10 min at room temperature, followed by incubation in 50 mM ammonium chloride buffer for 30 min, blocking in PBS containing 1% BSA for 1 h, and permeabilization in 0.5% saponin, 0.1% BSA in Tris buffered saline (TBS) for 30 min. The following primary antibodies were used: mouse monoclonal anti-annexin A2 1G7 (10 µg/ml) (Novus Biologicals), mouse monoclonal anti-paxillin (1:100) (BioLegend), rabbit polyclonal anti-caveolin 1 (1:200) (Abcam ab2910) for 16 h at 4 °C. Specific signals were revealed with the following secondary antibodies: goat anti-mouse-Cy3 (1:1,000) or Alexa 647 (1:200), goat anti-mouse-FITC (1:1,000), rabbit anti-mouse-FITC (1:1,000) (Jackson Immunoresearch Laboratories, West Grove, PA) for 1 h at room temperature. Biotin-conjugated peptides were visualized by incubation with avidin-FITC (5 µg/ml) (Invitrogen). For annexin A2/paxillin co-staining, cells were first incubated with the anti-annexin antibody overnight at 4 °C, followed by Alexa 647-conjugated goat anti-mouse secondary antibody for 1 h at room temperature; after extensive washing, cells were then incubated with the anti-paxillin antibody for 1 h at room temperature, followed by FITC-conjugated rabbit anti-mouse antibody for 1 h at room temperature. Actin was revealed by incubation with 5 units of rhodamine-phalloidin (Thermo Fisher Scientific) for 20 min at room temperature. Coverslips were mounted onto microscope slides in Vectashield (VectorLab) mounting media containing DAPI for nuclei staining. Images were acquired with a Leica TCS S8 confocal microscope (Leica Microsystems) and pixel co-localization was analyzed with Fiji ImageJ software^47^.

### Western Blot

KS1767 cells were seeded (1 × 10^6^) into 6 well plates and grown to 80% confluence. Cells were incubated with each peptide (100 µM) for 10, 30 and 60 min, followed by protein extraction in 50 mM Tris, 150 mM NaCl, 1% NP-40, phosphatase/protease inhibitors (Roche). Protein extracts were resolved by SDS-PAGE, transferred to nitrocellulose membranes and decorated with standard protocols. The following primary antibodies (all Cell Signaling) were used at a 1:1,000 dilution: rabbit polyclonal anti-Fak (#3285), rabbit monoclonal anti-phosphorylated Fak (Tyr^397^, #8556), mouse monoclonal anti-Akt (2H10), rabbit monoclonal anti-phosphorylated Akt (Ser^473^, D9E), rabbit monoclonal anti-phosphorylated p44/p42 (Thr^202^/Tyr^204^), and mouse monoclonal anti-Erk1/2 (p44/p42) (3A7). The rabbit anti-actin antibody (Sigma) was used at a 1:2,000 dilution.

### Annexin A2 silencing

Silencing of annexin A2 expression was performed in KS1767 cells seeded into 6-well plates. Cell transduction was obtained with four alternative lentiviral shRNA clones directed against human annexin A2 (pGFP-C-shLenti, Origene Technologies). A scrambled shRNA lentivirus was used as a negative control. GFP-positive cells were sorted with a sy3200 Cell Sorter (Sony Biotechnology) and seeded into 6-well plates. Cell extracts were obtained in 50 mM Tris, 150 mM NaCl, 1% NP-40, phosphatase/protease inhibitors (Roche). To evaluate the downmodulation of annexin A2 protein, a Western blot was performed with a mouse monoclonal anti-annexin A2 (Novus Biologicals).

### Cell adhesion, migration and viability/proliferation assays

For cell adhesion assays, 48-well flat-bottom plates were coated with 100 µl of fibronectin (20 µg/ml) or vitronectin (10 µg/ml) in PBS overnight at 4 °C. Wells were washed twice with PBS and blocked with PBS containing 1% BSA for 2 h at room temperature. Suspended cells were preloaded with each peptide for 30 min at 37 °C, followed by incubation onto the coated wells (50,000 cells/well in 200 µl complete medium) at 37 °C for 1 h. Non-adherent cells were removed by extensive washing with PBS. Adhered cells were counted under a light microscope (10 random fields/experimental point). For cell migration assays, 1 × 10^5^ KS1767, KRIB or B16F10 cells were applied to the upper chamber of transwell inserts (Costar) in the presence of each peptide at 100 µM; LLC cells were incubated with 10 µM of the peptides. After 24 h, migrated cells were fixed, stained in crystal violet (Fisher) and counted (5 fields/well) under a light microscope. For the *in vitro* wound healing assays, cells were grown to confluence in 6-well plates, at which point a scratch (“wound”) was created with a pipetman tip. Cells were washed and incubated with either medium only, or each synthetic peptide at the indicated concentrations. Wounds were imaged under a phase contrast microscope at the beginning of the incubation and after 16 h, followed by scoring of the degree of closure. Cell viability/proliferation was evaluated by WST assay (Sigma Aldrich) in KS1767 cells grown in 48-well plates and maintained for 24, 48, 72 or 96 h in DMEM supplemented with 10% FBS plus each test peptide.

### Tumor models

KRIB and B16F10 cells were incubated for 30 min with either pen, LGRFYAASG-pen, or LGRFYAASG at 100 µM, and LLC cells were incubated with the same peptides at 10 µM, followed by washing with non-supplemented DMEM. Cohorts of C57BL/6 females mice (n = 8), 8–12-weeks-old, were administered intravenously with 1 × 10^5^ KRIB or B16F10 cells, or 1.5 × 10^5^ LLC cells. After 8 weeks for KRIB cells, 21 days for B16F10 cells, and 13 days for LLC cells, lungs were removed, rinsed with PBS, and weighed. Lung lobes were dissociated and the number of tumor foci (dark color) was determined at both dorsal and ventral positions under a binocular dissection microscope for B16F10 cells. For KRIB and LLC cells, lungs were fixed in Bouin’s fixative (Sigma-Aldrich) overnight at room temperature. The following day, lungs were washed with 70% ethanol and tumor foci determined under a binocular dissection microscope. Five micron tissue sections were cut in a Microm HM 325 Rotary Microtome (Thermo Scientific, Walldorf, Germany) and stained with H&E, followed by image acquisition with a Nikon Ti-S Inverted Microscope (Nikon Instruments). The Institutional Animal Care and Use Committees (IACUCs) of both the University of Texas M. D. Anderson Cancer Center and the University of New Mexico health Sciences Center approved all animal experiments. All procedures reported in this paper were conducted in full compliance with the indications from the corresponding IACUCs, which follow the standard guide care and use of laboratory animals.

### Bioinformatics and statistics

Ingenuity Pathway Analysis (IPA) software (Qiagen) was used to analyze protein-protein interactions with the canonical actin cytoskeletal signaling pathway. The assessment of statistical significance between groups was carried out according to one-way ANOVA test with the GraphPad Prism software, the level of statistical significance defined as a value of *P* ≤ 0.05.

## Electronic supplementary material

Supplementary Information
