# Supplementary material for: Intracellular targeting of annexin A2 inhibits tumor cell adhesion, migration, and in vivo grafting
Source: Sci Rep. 2017 Jun 26;7:4243. doi: 10.1038/s41598-017-03470-w (PMC5484684; doi:10.1038/s41598-017-03470-w)
Supplement: Supplementary file 1 — Supplementary Information [file 41598_2017_3470_MOESM1_ESM.pdf]

Intracellular targeting of annexin A2 inhibits tumor cell adhesion, migration, and *in vivo* grafting

Daniela I. Staquicini, Roberto Rangel , Liliana Guzman-Rojas, Fernanda I. Staquicini, Andrey S. Dobroff, Christy A. Tarleton, Michelle A. Ozbun, Mikhail G. Kolonin, Juri G. Gelovani, Serena Marchiò, Richard L. Sidman, Katherine A. Hajjar, Wadih Arap, Renata Pasqualini

Supplementary information

Table S1. Mass spectrometry analysis of protein candidates isolated from fraction F46.

Mass-spec retrieved peptides	Position on the protein (residues)	Protein name	Protein ID
GFSVVADTPELQR	96-109	LASP1	NP_006139.1
TQDQISNIK	112-120		
YHEEFEK	122-128		
FITHAPPGEFNEVFNDVR	20-37	CAPZA1	NP_006126.1
LLLNNDNLLR	38-47		
EASDPQPEEADGGLK	103-118		
FTITPPTAQVVGVLK	179-193		
DVQDSLTVSNEAQTAK	211-238		
QDIAFAYQR	87-95	annexin A2	AAH66955.2
TPAQYDASELK	123-133		
TNQELQEINR	154-163		
RAEDGSVIDYELIDQDAR	197-214		

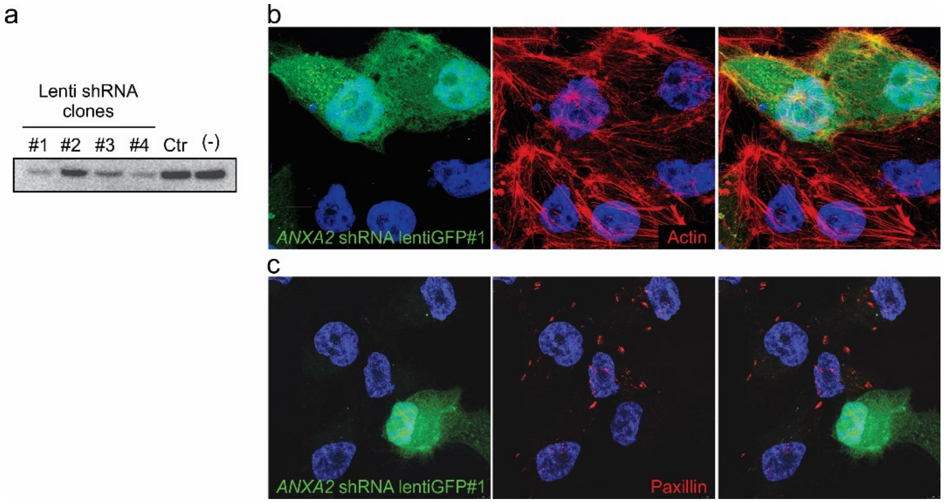

Figure S1. Silencing of annexin A2 disrupts the organization of actin filaments and focal adhesions. (**a**) The expression of annexin A2 was silenced in KS1767 cells by transduction with four alternative lentiviral shRNA (clones #1-4) against human annexin A2. A scrambled shRNA was used as a negative control (Ctr); (-), no lentivirus. GFP-positive (shRNA-transduced) cells were sorted and grown into 6-well plates to 80% confluence. Cell were lysed and protein extracts were separated by SDS-PAGE, blotted to nitrocellulose membranes and decorated with a specific anti-annexin A2 antibody. (**b, c**) Pools of cells infected with the best performing lentiviral shRNA clone (#1) were grown overnight onto circular coverslips coated with fibronectin and vitronectin, fixed and stained for IF and confocal imaging. Green, shRNA-transduced cells; (**b**) red: actin (rhodamine-phalloidin); (**c**), red: paxillin (Alexa 647 secondary antibody). Scale bar, 10 μm.

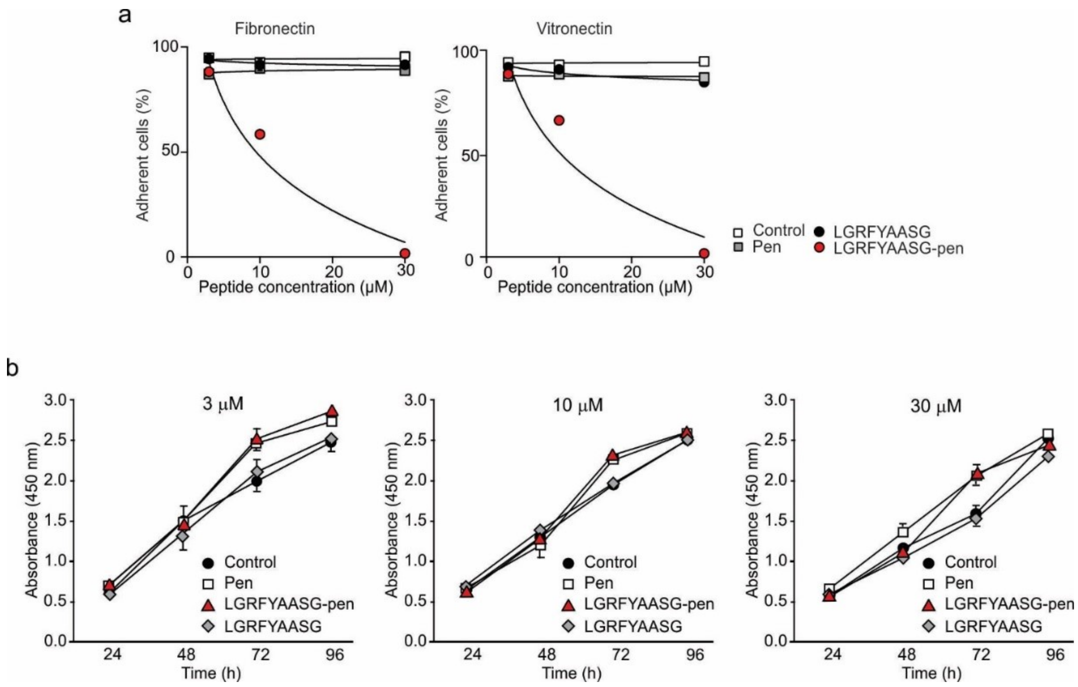

Figure S2. Tumor cell migration and viability assays. (**a**) KS1767 cells were exposed to increasing peptide concentrations (3-10-30 μM), followed by evaluation of cell adhesion to different matrix proteins (**b**) or proliferation at different time points by WST assay. Bars represent mean ± SEM of triplicate experimental points.

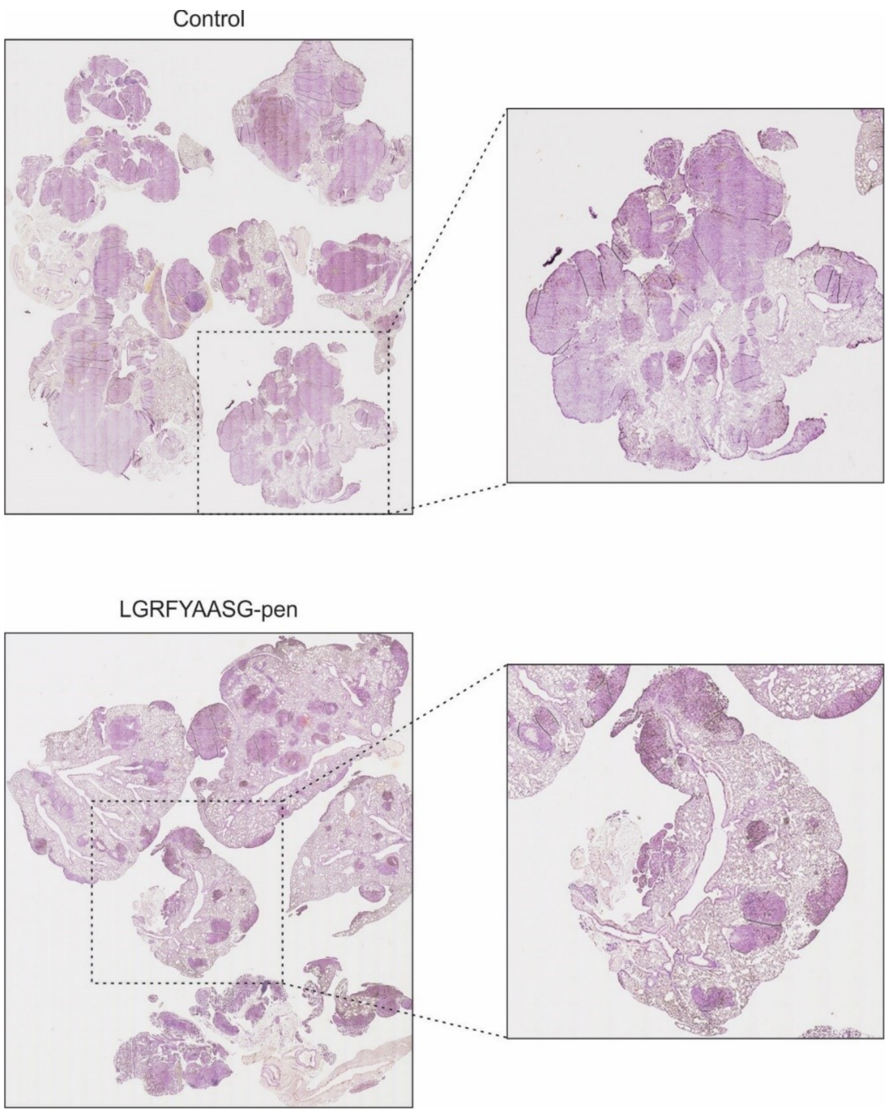

Figure S3. Pathological analysis of lungs in tumor-bearing mice. Representative histological analysis of tumor foci in lungs from animals administered with B16F10 cells. Slide sections were obtained from formalin-fixed and paraffin-embedded tissue (5-µm slices) and stained with H&E.
